# Transverse Myelitis in Naloxone Reversible Acute Respiratory Failure—A Case Report

**DOI:** 10.21980/J8B659

**Published:** 2022-10-15

**Authors:** Chance Dodson, Joshua Gentges

**Affiliations:** * University of Oklahoma, Department of Emergency Medicine, Tulsa, OK

## Abstract

Transverse myelitis (TM) is a rare inflammatory myelopathy presenting as bilateral neurologic deficit localized to the spinal cord. A critical management step in the emergency department (ED) is evaluating for and treating acute reversible causes such as mass lesion or reversible ischemia when present. Described in this case report is TM presenting after a respiratory arrest in suspected opioid overdose. Magnetic resonance imaging (MRI), ideally with contrast, and lumbar puncture are essential diagnostic studies to confirm inflammation. Finally, further diagnostic efforts are aimed at evaluation and treatment for other concurrent illnesses.

**Topics:**

Transverse myelitis, transverse myelopathy, hypoxia, opioid overdose, hypoxic spinal cord injury.

**Figure:**
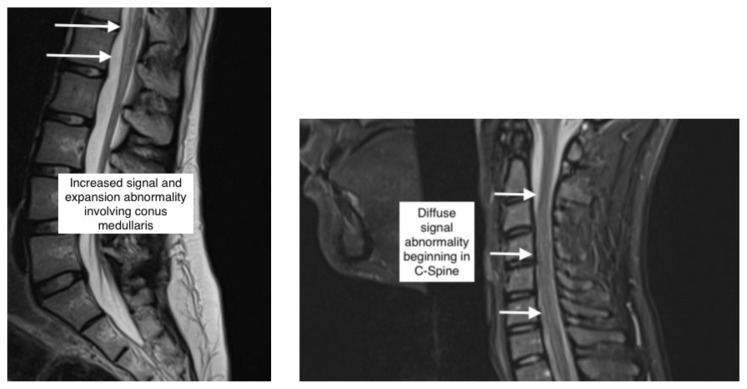


## Brief introduction

Transverse myelitis (TM) is a challenging diagnosis in the emergency department (ED). The presentation is varied, and practical application of diagnostic criteria incorporating clinical findings, imaging and laboratory studies is often inconsistent.^1^ Further, diagnostic criteria for other specific myelopathies overlap with TM.^1^,^2^,^3^ Transverse myelitis classically presents as abrupt motor, sensory and/or autonomic deficit correlating to a specific spinal cord level. It can be a primary idiopathic process or secondary to other disease processes, including systemic inflammatory disease, paraneoplastic syndrome, or neurologic inflammatory disorder. The ED diagnosis in clinical practice is often made while imaging, cerebrospinal fluid (CSF) studies, and other serologic tests are incomplete. Identification of associated disease process can be delayed for over 9 months.^4^ The diagnosis of primary idiopathic TM ranges between 16–60% of cases.^5^ In more recent years, the term TM is classified under the more general term transverse myelopathy, and specifies a lesion due to an inflammatory process. An essential step in the diagnosis of TM is exclusion of other spinal cord injury or compressive spinal lesion that may be amenable to specific medical or surgical intervention.

A more frequently encountered ED diagnosis is opioid overdose, which is now even more common in the wake of the COVID-19 pandemic.^6^ Treatment in the field with an opioid antagonist is often safely initiated by bystander or emergency medical services (EMS).^7^,^8^ While there are reports of TM associated with intranasal and IV heroin use, there are no reports to this author’s knowledge of TM associated with synthetic opioids by any route.^9^,^10^ We present a unique case of TM presenting as transient paraplegia following administration of naloxone in the field for presumed opioid overdose.

## Presenting concerns and clinical findings

An 18-year-old female with history of spinal epidural anesthesia for vaginal delivery 9 months prior and history of syncope presented by ambulance for apnea that resolved following intranasal naloxone. The patient had a witnessed loss of consciousness with initiation of immediate bystander CPR. Paramedics on scene noted apnea and cyanosis on their arrival. The patient improved and was awake and fully oriented on arrival to the ED. Exam on presentation revealed bilateral arm weakness and complete paralysis of lower extremities. Fine touch and position sense were intact with loss of pain perception. Her symptoms rapidly improved to the point that she ambulated independently within one hour of arrival. The patient did report taking a “blue pain pill” from a pill bottle belonging to a recently deceased family member just prior to symptom onset. She endorsed back pain that had been present for the last six months. It worsened following a low speed motor vehicle accident three months preceding presentation, and acutely worsened three days prior to ED presentation without any additional trauma.

## Significant findings

Magnetic resonance imaging of the brain, cervical, thoracic and lumbar spine without contrast was obtained and revealed increased signal throughout the spinal cord from C-1 to the conus medullaris with mild expansion consistent with transverse myelitis. Neurosurgery was consulted and the patient was treated with 200 mg intravenous (IV) dexamethasone in the ED and a lumbar puncture was performed. Results of CSF analysis included WBC count of 2 cells, 100% lymphocytes, Protein 23 mg/dl and glucose 59 mg/dl. Urine drug screening was negative including screen for opiate, tetrahydrocannabinol, phencyclidine. Of note, the drug screen performed at this facility was not sensitive for synthetic opioids such as oxycodone and fentanyl.

## Patient course

No focal neurological deficits recurred during the hospital course. She received a 5-day course of methylprednisolone 125 mg IV q 6 hrs. She did have new onset of chest pain attributed to injury from bystander chest compressions. A migraine headache was treated with sumatriptan during hospitalization. Her hospital course was complicated by symptomatic bradycardia with heart rates in the 30’s. Electrophysiology was consulted and sumatriptan was discontinued followed by resolution of bradycardia.

## Discussion

A broad differential diagnosis was considered, and appropriate diagnostic studies were obtained leading to timely diagnosis, appropriate specialist consultation, and medical management with corticosteroids. Additional diagnostic studies including MRI with gadolinium contrast, specialized CSF studies, and serum studies to search for underlying systemic and inflammatory disease could have further solidified the diagnosis of idiopathic TM or suggested other possible primary disease processes.

Diagnostic criteria for specific myelopathies based on underlying etiology and application of criteria vary in clinical practice. The Transverse Myelitis Consortium Working Group in 2002 proposed a standardized diagnostic approach for TM to provide groundwork for clinical practice and clinical trials.^1^ A follow up study in 2018 found that nearly 70% of patients referred to Mayo Clinic neurology with a working diagnosis of idiopathic TM likely had an alternative myelopathy according to the above criteria, the most common being multiple sclerosis.^4^ This finding highlights the importance of initiating a standardized evaluation of myelopathy in the ED and consultation to reveal associated disease in the work up of TM, as therapies targeted to associated disease processes will likely lead to improved outcomes.

In this case, the patient had a witnessed, hypoxic episode and inflammatory findings detected on non-contrast MRI. Early physical exam raised concern for anterior cord syndrome given loss of motor function and fine touch with preservation of pain sense. However, no compressive lesion was found, and a focal anterior spinal artery lesion was considered unlikely given rapid resolution of paraplegia. Further, distribution of MRI findings were not limited to the perfusion territory of the anterior spinal artery. The brief time span from onset to resolution of symptoms was atypical for TM. According to the above criteria, symptom onset to clinical nadir in acute TM occurs between 4 hours and 21 days. Further, according to the consortium publication, inflammation cannot be definitively confirmed without a gadolinium-enhanced MRI. The patient’s combination of rapid improvement with MRI findings concerning for inflammatory process could be explained by reversible systemic hypoxia imposed on subacute, high spinal cord myelitis. The patient’s neurologic deficits were preceded by several days of acute on chronic back pain. Immediately prior to onset of patient’s apnea and cyanosis, she ingested a “blue pain pill.” While oxycodone is produced legitimately as a blue pill, counterfeit oxycodone pills often have the same color and shape and may contain fatal doses of fentanyl in a single pill.^11^ However, a brief period of systemic hypoxia alone did not fully explain the patient’s focal deficits and MRI findings because other end organ damage would be expected were this the case. It is known that recrudescence of neurologic deficits from prior stroke does occur in the setting of hypoxia and other metabolic derangement.^12^ This has also been reported in the setting of isolated exposure to opioids even in the absence of hypoxia and other metabolic derangement.^13^ It is possible that acute toxic and metabolic insult from opiate exposure and hypoxia fully expressed a pre-existing subclinical myelitis in this patient.

Transverse myelitis should be suspected in any patient presenting with bilateral neurological deficit localizable to a spinal level. Immediate ED management requires exclusion of reversible causes amenable to neurosurgical or other acute intervention and initiation of high dose corticosteroids. Specialist consultation is required for further treatment of TM and to coordinate further evaluation for possible concurrent illness. This case of transverse myelitis in the setting of presumed opioid overdose highlights the importance of careful consideration of atypical presentations of classical disease processes and the possibility of coexisting pathology to develop appropriate differential diagnoses, diagnostic studies, and treatment plans.

## Supplementary Information








