# Supplementary figures and images for: Transverse Myelitis in Naloxone Reversible Acute Respiratory Failure—A Case Report
Source: J Educ Teach Emerg Med. 2022 Oct 15;7(4):V15–8. doi: 10.21980/J8B659 (PMC10332668; doi:10.21980/J8B659)

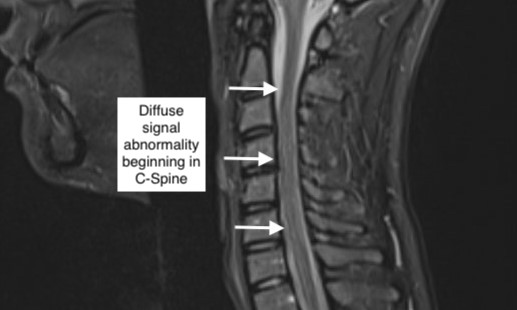

Supplement: Supplementary file 1 [file JETem-7-4-V15-supp1.jpeg]

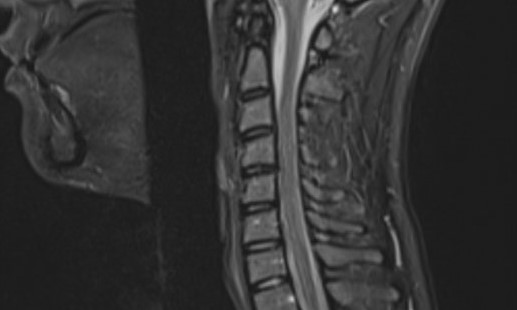

Supplement: Supplementary file 2 [file JETem-7-4-V15-supp2.jpg]

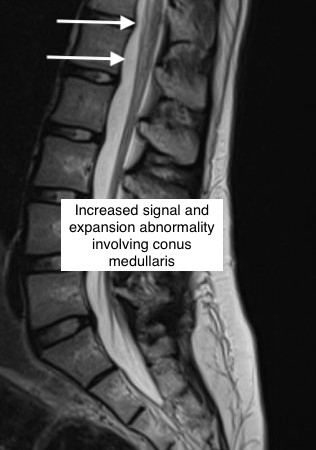

Supplement: Supplementary file 3 [file JETem-7-4-V15-supp3.jpg]

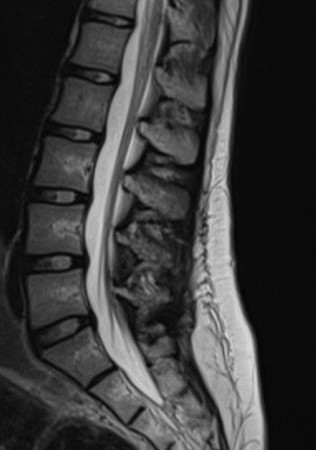

Supplement: Supplementary file 4 [file JETem-7-4-V15-supp4.jpg]
